# Substrate recognition and cleavage-site preferences of Lon protease

**DOI:** 10.1016/j.jbc.2025.108365

**Published:** 2025-02-27

**Authors:** Melanie Cragan, Neha Puri, A. Wali Karzai

**Affiliations:** Graduate Program in Molecular and Cellular Biology, Department of Biochemistry and Cell Biology, Center for Infectious Diseases, Stony Brook University, Stony Brook, New York, USA

**Keywords:** ATPases associated with diverse cellular activities (AAA+), protein homeostasis, Lon protease, proteolysis, substrate specificity

## Abstract

The evolutionarily conserved AAA+ Lon protease plays a pivotal role in protein homeostasis by precisely remodeling the proteome and specifically removing unfolded, damaged, and surplus natively folded regulatory proteins. Proteolysis by Lon comprises the three fundamental stages of substrate recognition *via* specific amino acid sequence motifs (degrons), ATP-fueled substrate unfolding and translocation into a sequestered proteolytic chamber, and cleavage of the translocated polypeptide by the peptidase domain. Although a plethora of Lon substrates have been identified in several bacterial species, broadly applicable rules that govern recognition of numerous substrates, and hence the ability to *de novo* identify new Lon substrates and regulatory pathways, has lagged behind. Similarly, cleavage-site preferences of Lon proteases, and whether these crucial enzymes from diverse bacterial species share similar preferences, have remained underexplored. In this study, we report the identification and characterization of a class of high-affinity autonomous C-terminal *Yersinia pestis* Lon recognition degrons, variants of which are present in numerous known and new *Yersinia pestis*-Lon substrates and broadly distributed in diverse bacterial species. Moreover, the identification of this degron group offers the predictive power to discover new Lon substrates in eubacteria. Furthermore, cleavage-site preference analyses of multiple Lon substrates reveal that the Lon peptidase domain preferentially cleaves translocated polypeptides after phenylalanine residues to generate peptides that range from 7 to 35 residues, with an average length of 11 residues, a general feature conserved among Lon proteases from phylogenetically distinct bacterial species.

Protein degradation plays a central role in the maintenance of ideal concentrations of key regulatory proteins and the disposal of surplus, damaged, or misfolded proteins. In bacteria, AAA+ proteases Lon, ClpXP, ClpAP, HslUV, and FtsH play a fundamental role in regulating cellular responses to key physiological transitions and ridding the cell of unwanted regulatory proteins (1, 2). The Lon protease is conserved from bacteria to humans and plays a major role in fundamental cellular processes that include protein homeostasis, stress responses, and bacterial pathogenesis (1, 3, 4, 5, 6, 7, 8, 9, 10, 11, 12, 13, 14, 15, 16, 17, 18, 19, 20, 21, 22, 23, 24, 25, 26, 27, 28, 29, 30). Lon forms a ring-shaped homo-hexamer, and each Lon monomer contains an N-terminal substrate recognition domain, a central ATPase domain involved in substrate unfolding and translocation, and a C-terminal peptidase domain that harbors the active site serine-lysine catalytic dyad. The Lon protease is thus endowed with all the necessary attributes to form a hexameric chambered protease capable of the specific recognizing, unfolding, and degrading a broad range of proteins.

The capacity of a cell to remodel its proteome relies on the exquisite substrate specificity of its proteolytic complement. Existing evidence suggests that substrate recognition depends on intrinsic amino acid sequence signals (degrons) located at the amino, carboxyl, or internal segments of protein substrates (31). Lon recognition motifs (degrons) are either constitutively available for protease binding or become conditionally exposed upon changes in growth environment, dissociation of interacting partners, unfolding, or following primary cleavage events (32). The prevailing hypothesis is that Lon recognizes sequences motifs rich in hydrophobic residues that become accessible in unfold polypeptides (33). However, it is becoming increasingly clear that Lon also recognizes a growing number of native folded proteins carrying specific recognition motifs or degrons. To date, only a handful of individual Lon recognition degrons have been characterized, including the 11 residue ssrA tag (23, 24, 34), the C-terminal 20 residues of SulA (35), an internal sequence (residues 49–68) of β-galactosidase called β20, the N-terminal sequence (residues 1–21) of SoxS (36), the N-terminal proximal sequence (residues 12–31) of UmuD (37), and the C-terminal 20 residues of HspQ and Y2853 (2). However, broadly applicable rules, that is, the identity of degrons that are present in numerous known and new substrates and govern autonomous substrate selection by Lon, have not been elucidated. This paucity of information on broadly applicable substrate recognition preferences of Lon protease is due largely to the fact that many Lon substrates are often studied individually and in insufficient details. Consequently, it has been challenging to decipher sequence rules governing substrate selection by Lon or generally predict and characterize pathways in which Lon plays an important role.

We recently identified close to 90 putative new *Yersinia pestis* (yp)-Lon substrates, using a global Lon-specific proteomics approach in yp during induction of the type III secretion system, a key physiological transition that requires precise remodeling of the bacterial proteome (2). The availability of this large set of yp-Lon substrates presented the unique opportunity to systematically enhance our understanding of Lon protease substrate and cleavage-site preferences. In this study, we elucidated the substrate recognition and cleavage-site preferences of yp-Lon protease and present evidence on the preferences of Lon proteases from yp*, Escherichia coli*, and *Mycoplasma pneumoniae* (MP). Our findings unveil the cleavage-site preferences of the *Yersinia* Lon protease and reveal a high degree of conservation in the preferences of Lon proteases from these diverse bacterial species. Most significantly, we report the identification and biochemical characterization of an autonomous and broadly distributed Lon recognition degron group that is present in many known and new yp-Lon substrates and facilitates the *de novo* identification of new Lon targets in diverse bacterial species.

## Results

### Identification of a widely distributed yp-Lon recognition degron

**Figure 1:**
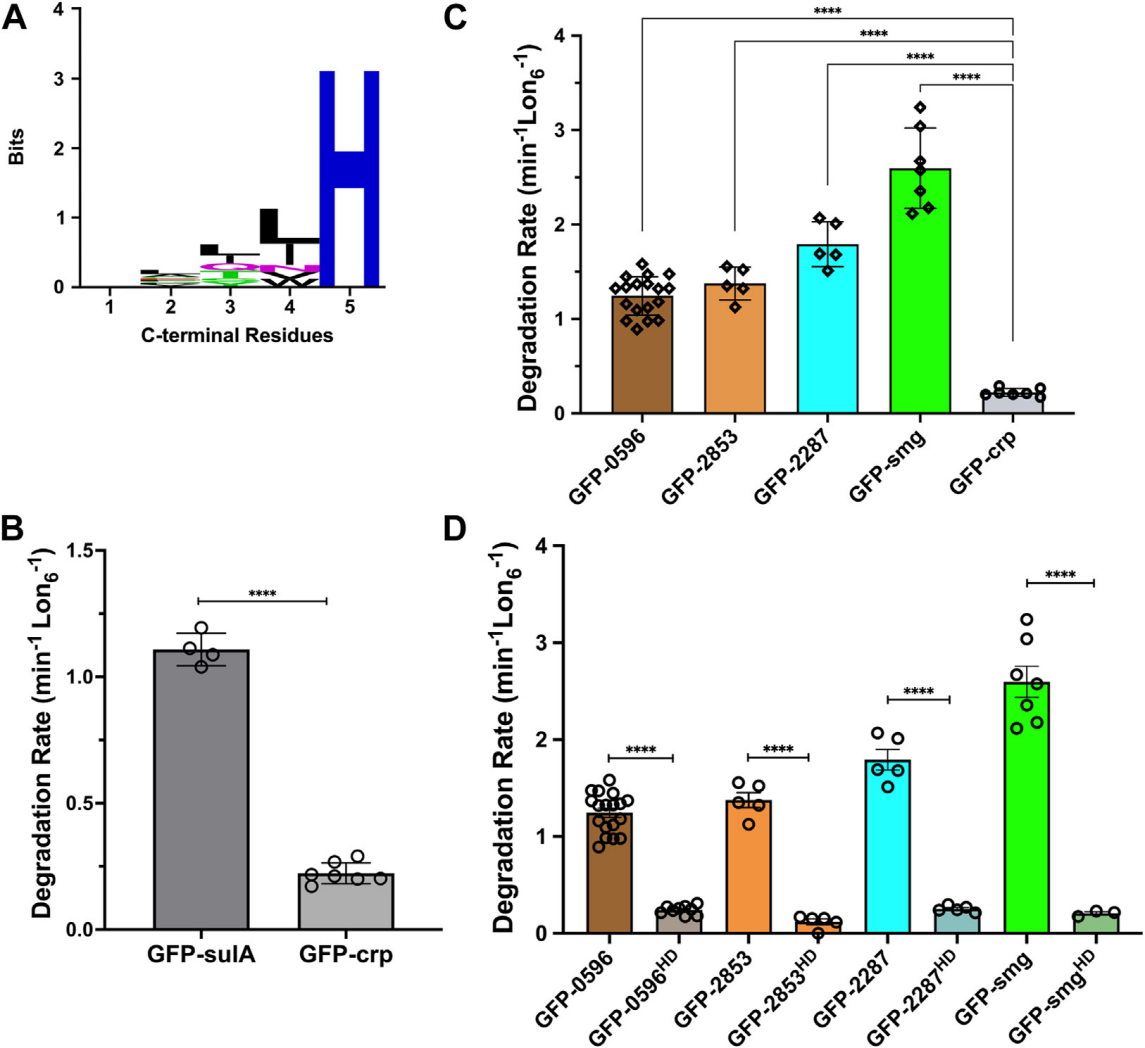
**C-terminal sequences of several Lon^TRAP^ substrates serve as autonomous Lon degron.***A,* Weblogo (http://weblogo.berkeley.edu/logo.cgi) analysis of the C-terminal sequence motif found in nine Lon^TRAP^ substrates highlight the invariant terminal histidine residue. *B,* the C-terminal 19 residues of *Yersinia* SulA (sulA20), degron as a positive control, and the C-terminal 25 residues of the Lon^TRAP^ substrate Crp, as a negative control, were appended to the GFP reporter and their propensity to serve as autonomous Lon recognition degron were examined in an *in vitro* degradation assay. *t* test analysis was performed to analyze the data, yielding *p* value of <0.0001. *C,* the C-terminal 20 residues of 4 Lon^TRAP^ substrates were appended to the GFP reporter and their propensity to serve as autonomous Lon recognition degron were examined in an *in vitro* degradation assay. One-way ANOVA analysis, with multiple comparisons, was performed to analyze the data, comparing each substrate to the GFP-crp control. *p* values were <0.0001. *D,* degradation rates of degron-tagged GFP reporter and the H286D mutants of these reporters were determined and plotted to access the importance of the C-terminal histidine residue. Data presented in all graphs are from more than three independent experiments (mean ± SD). *p* < 0001 = ∗∗∗∗.

Lon protease serves as a principal proteolytic component in many bacterial species and as such plays a pivotal role in general cellular physiology, protein quality assurance, stress response, and bacterial pathogenesis (21). To gain deeper insight into the role of Lon protease in bacterial pathogenesis, we used the Lon^TRAP^ approach and identified close to 90 *in vivo Yersinia* Lon (yp-Lon) substrates and regulators during a key physiological transition, the induction of type III secretion system (2). We anticipated that sequence analysis of numerous native substrates might yield insights into substrate recognition rules for the *Yersinia* Lon protease, and perhaps Lon proteases in general. Unfortunately, use of various sequence analysis approaches yielded no clear and discernable common recognition motifs among these substrates. Nonetheless, biochemical characterization of a captured native substrate, Y2853, revealed the presence of an autonomous high-affinity C-terminal Lon recognition degron (2). Intriguingly, a subset (10%) of Lon^TRAP^ substrates carried sequence variants of this C-terminal motif, with the x–L/I–L/I/V–H sequence motif (Fig. 1*A*), invariably ending with a terminal histidine residue (Table 1). Notably, a similar autonomous motif is present at the C terminus of SulA, a known *E. coli* Lon (Ec-Lon) substrate (38, 39, 40).

**Table 1:** List of Lon substrates, their gene ID, and putative degrons sequences

Lon substrates	YPO CO92 ID	C-terminal degron
Smg (Lon^TRAP^)	YPO 0244	ENAYKQMEELLFEVNDGLYH
2287 (Lon^TRAP^)	YPO 2020	PIAEMIKMQIHSIEQQRVTLH
0596 (Lon^TRAP^)	YPO 0337	ISESSCFGPDRKKHKFTVH
2853 (Lon^TRAP^)	YPO 1330	IGNNDEEEAPLTATSYPIIH
SulA (known Lon degron)	YPO1436	TKVGSGQCATLKIHSYLYH
Crp (Lon^TRAP^, lacking H-degron)	YPO0175	MLEDQNLISAHGKTIVVYGTR
ApaG (predicted)	YPO 02220	GGQAFRTVIPVFRLAIPALILH
2220 (predicted)	YPO 0491	PIDYFKSDAEEYDLSLH

This observation led to the hypothesis that this motif might exemplify a widely distributed group of C-terminal Lon recognition degrons present in numerous known and new Lon substrates. To determine whether variants of this C-terminal motif are necessary and sufficient for recognition by Lon when appended to unrelated reporter proteins and convert them into Lon substrates, that is, serve as autonomous Lon recognition degron, we selected four representative members of this group (Table 1) and generated fluorescent reporter constructs where approximately 20 C-terminal residues of each putative degron were appended to the C terminus of a circularly permuted GFP variant, CP6 (41, 42), hereafter referred to as GFP. It has been demonstrated that Lon requires the presence of a C-terminal recognition sequence to degrade this GFP variant (42). We first used the C-terminal 20 residues of yp-SulA, a known Lon recognition degron, to generate the GFP-sulA reporter as a positive control, and the C-terminal 20 residues of *Yersinia* Crp (GFP-crp), a Lon^TRAP^ substrate lacking the C-terminal H degron, as a negative control (Table 1).

We cloned, expressed, and purified all six GFP-degron reporters and assessed their propensity to serve as Lon substrates in an *in vitro* proteolysis assay, designed to test the ability of Lon protease to recognize and degrade substrates (2). This analysis showed that the positive control GFP-sulA reporter is recognized and degraded efficiently by Lon (Fig. 1*B*), at a degradation rate (*K*_*DEG*_ = 1.1 min^−1^ Lon_6_^−1^) that is significantly faster than the negative control GFP-crp (*K*_*DEG*_ = 0.2 min^−1^ Lon_6_^−1^) (Fig. 1*B*). These data suggested that the GFP-degron reporters are well suited for assessing how well Lon recognizes members of this Lon^TRAP^ degron group. Analysis of the GFP reporter carrying variants of this degron group from four Lon^TRAP^ substrates showed that Lon degrades these substrates with a range of degradation rates. GFP-smg exhibited the fastest degradation rate (*K*_*DEG*_ = 2.6 min^−1^ Lon_6_^−1^), whereas GFP-0596 exhibited a modest degradation rate (*K*_*DEG*_ = 1.2 min^−1^ Lon_6_^−1^), as compared to the positive and negative controls (Fig. 1, *B* and *C*). Notably, the GFP-sulA positive control was degraded at rate comparable to the slowest Lon^TRAP^ reporter, GFP-0596 (Fig. 1*C* and Table 2). Given that all the examined GFP reporters have the same intrinsic thermodynamic stability, differences in the observed degradation rates are likely indicative of the relative affinity of Lon for each degron variant. Together, these data suggest that all examined substrates carrying variants of this C-terminal H degron group are recognized and degraded by Lon and serve as autonomous Lon recognition motifs.

**Table 2:** Degradation rates and relative affinities of the Lon degrons and their variants

Substrate	*K*_*DEG*_ ± SD (min^−1^ hexamer^−1^)^a^	*K*_*D*_ ± SD (μM)^a^
GFP-ApaG	1.4 ± 0.4	3.8 ± 0.2
GFP-2220	0.6 ± 0.08	30 ± 3
GFP-0596	1.2 ± 0.2	2.0 ± 0.3
GFP-2853	1.4 ± 0.2	8.5 ± 1.4
GFP-2287	1.8 ± 0.2	3.1 ± 0.5
GFP-smg	2.6 ± 0.4	0.24 ± 0.04
GFP-smg^HA^	0.3 ± 0.1	ND^b^
GFP-smg^LA^	0.6 ± 0.04	ND^b^
GFP-smg^YA^	0.9 ± 0.1	ND^b^
GFP-smg^YLH/AAA^	0.3 ± 0.1	ND^b^
GFP-smg^HD^	0.2 ± 0.03	BND^c^
GFP-2287^HD^	0.25 ± 0.03	BND^c^
GFP-ApaG^HD^	0.3 ± 0.03	BND^c^
GFP-2853^HD^	0.1 ± 0.07	BND^c^
GFP-2220^HD^	0.24 ± 0.19	BND^c^
GFP-0596^HP^	0.76 ± 0.08	ND^b^
GFP-0596^HD^	0.25 ± 0.04	ND^b^
GFP-0596^HR^	0.25 ± 0.07	ND^b^
GFP-0596^HC^	0.34 ± 0.04	ND^b^
GFP-0596^HS^	0.48 ± 0.02	ND^b^

a Values calculated from at least three independent repeats ± SD.

b Binding experiments not done (ND).

c Binding not detected (BND) and is outside of detectable range (>50 μM) under our assay conditions.

Numerous cytoplasmic bacterial proteases, in particular the Lon and ClpXP, disfavor C-terminal acidic residues in their recognition sequences (2, 24, 43, 44, 45). To further validate our findings and assess the importance of the C-terminal histidine residue in recognition by Lon, we generated GFP-degron mutants where the terminal histidine residue of each degron was changed to aspartate (referred to as HD mutants). We expressed and purified all GFP-degron^HD^ mutants and assessed their propensity to serve as Lon substrate in the *in vitro* degradation assay. This evaluation showed that mutation of the C-terminal histidine residue to aspartate drastically reduced degradation rates in all examined degrons (Fig. 1*D*), where all GFP-degron^HD^ mutants examined exhibited significantly diminished degradation rates of ≤0.3 min^−1^ Lon_6_^−1^. Collectively, these data support the conclusion that the C-terminal sequences of these proteins serve as autonomous Lon recognition degrons and that the terminal histidine residue is a principal determinant for recognition by Lon.

### Histidine is the ultimate preferred C-terminal residue for recognition by yp-Lon

We were curious to further explore the importance of the terminal histidine residue for recognition by Lon, fundamentally inquiring whether histidine is uniquely preferred by yp-Lon, or whether other residues serve better, or equally well, at the ultimate position of this degron. To facilitate this evaluation, we developed an unbiased *in vivo* assay to enable a high-throughput mutagenesis screen in which the degradation of the fluorescent GFP-degron reporter can be monitored. We selected the GFP-0596 reporter, which carries the Y0596 degron and exhibits a moderate degradation rate (Fig. 1 and Table 1), as a template to perform an extensive mutational analysis of the C-terminal histidine, substituting it to all other possible amino acid residues. We reasoned that GFP-degron reporters with C-terminal residues favored by Lon should be recognized better and proteolyzed faster and thus have shorter *in vivo* half-lives (t_1/2_)*,* whereas degron variants carrying terminal residues disfavored by Lon should be more stable and have longer t_1/2_.

To determine the effect of Lon on the *in vivo* stability of the reporter protein carrying this degron, we expressed the GFP-0596 degron construct in WT and *Δlon* cells, inhibited new rounds of protein synthesis, and monitored its stability over time. This analysis showed that GFP-0596 reporter was substantially more stable in cells lacking Lon protease (Fig. S1). We interpret these results to signify that the endogenous Lon protease plays a major role in the recognition and degradation of proteins carrying this degron *in vivo*.

**Figure 2:**
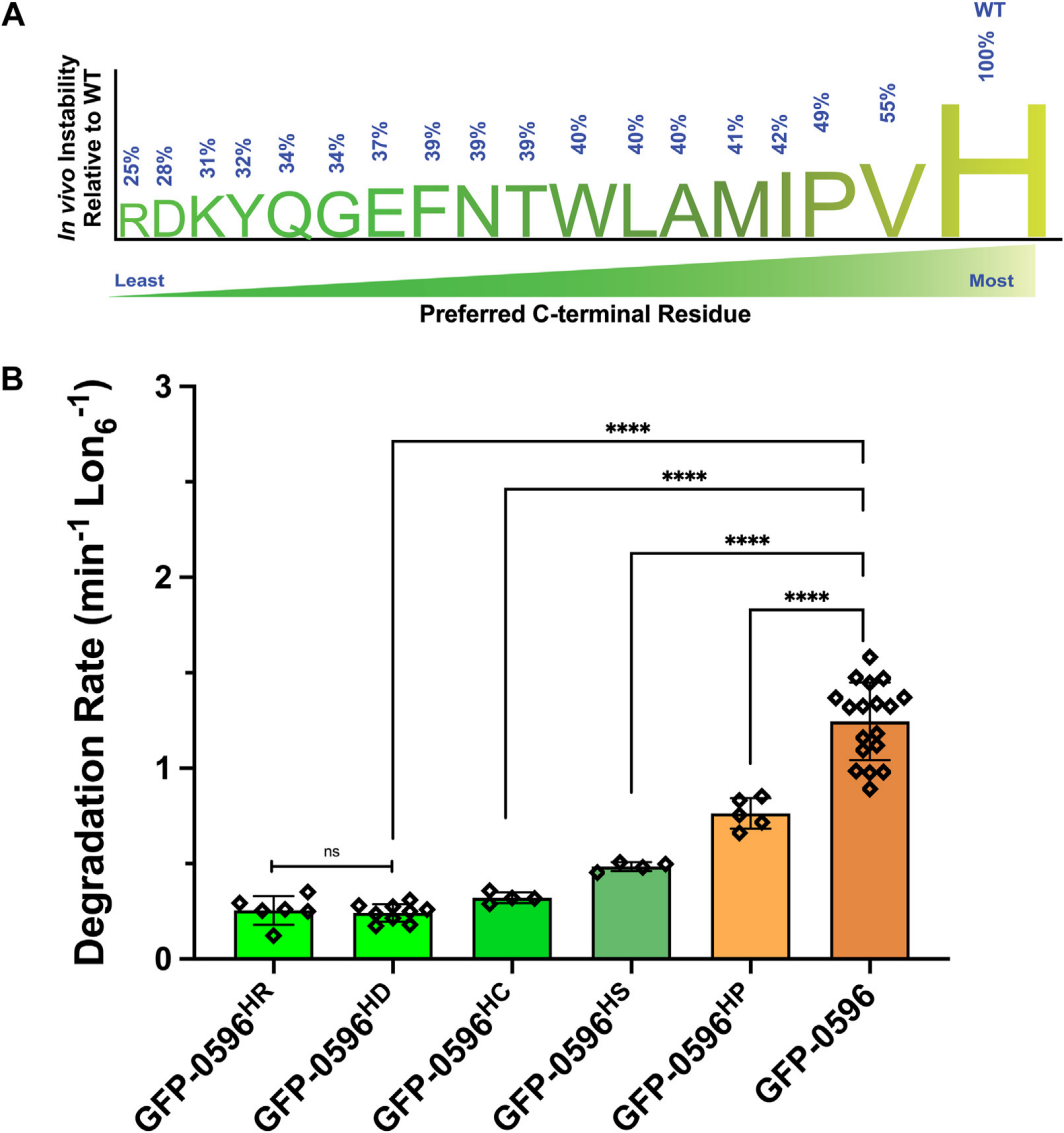
**Observed pattern of the *in vivo* and *in vitro* C-terminal residue preference of Lon protease.***A,* the *in vivo* degradation assays show that C-terminal histidine is strongly preferred by Lon protease. The degradation rate of the WT degron was set as 100% and the degradation rates of all other C-terminal residues were plotted relative to this standard. Degrons ending with C-terminal valine or proline residues ranked second (54%) and third (49%) best to histidine, respectively. Conversely, large or charged residues at the C terminus weaken Lon's propensity to recognize and degrade the reporter substrate. *B,* results of the *in vivo* mutant screen were verified by *in vitro* fluorescent-based degradation assays. Degradation rates of degron-tagged GFP-0596 and its HP, HS, HC, HD, and HR mutants were determined to demonstrate that the results of the *in vivo* screen can be validated *in vitro*. Data presented in graphs are from greater than three independent experiments (mean ± SD). One-way ANOVA, with multiple comparisons, was performed to analyze the data, comparing each substrate to the GFP-0596 control, yielding *p* values < 0.0001 (∗∗∗∗). Student *t* test analysis revealed no statistically significant difference (ns) between GFP-0596^HD^ and GFP-0596^HR^, with a *p* value of 0.3404.

Having established that Lon plays a key role in the disposal of the GFP-0596 reporter *in vivo*, we assessed the stabilities and t_1/2_ of 18 GFP-0596 degron mutants, where the terminal histidine was changed to all other amino acid residues, except serine and cysteine (Fig. 2 and Table S1). This analysis revealed the overall pattern of C-terminal residue preference of Lon (Fig. 2*A*). The WT Y0596 degron, with a terminal histidine residue, exhibited the fastest degradation rate with the shortest *in vivo* t_1/2_, demonstrating that Lon does indeed prefer a C-terminal Histidine residue, suggesting that nature has selected the most preferred C-terminal residue for recognition by Lon. Degron mutants with C-terminal valine or proline residues ranked second and third best, respectively, exhibiting approximately half the degradation rate compared to the WT degron. In contrast, mutants with C-terminal arginine or aspartate residues ranked among residues most disfavored by Lon (Fig. 2 and Table S1), exhibiting the highest stability and longest t_1/2_.

### The *in vivo* degron preferences of Lon are recapitulated *in vitro*

The *in vivo* degradation analysis carries the potential caveat that expression and stability of other cellular factors (including other proteases and adaptor proteins) might be impacted, which could have unintended effects on the apparent degradation rates of the GFP-degron mutants. Indeed, we observed a substantially slower disappearance of the GFP-0596 reporter in the *Δlon* cells (Fig. S1), suggesting that other cellular protease contribute, albeit to a much lower extent, to degradation of this substrate in the absence of Lon protease. To address this limitation and validate the outcome of the *in vivo* studies, we purified select GFP-degron variants with either a relatively high (H286R, and H286D) or a low (H286P) *in vivo* stability and measured their degradation rates *in vitro*. We also evaluated the degradation rates of the H286C and H286S variants of this degron, which were not recovered in the *in vivo* stability analysis described above. We observed that the H286P mutant had an *in vitro* degradation rate (*K*_*DEG*_) of 0.7 min^−1^ Lon_6_^−1^, nearly half the rate of the WT histidine containing degron (*K*_*DEG*_ = 1.2 min^−1^ Lon_6_^−1^), and the H286D mutant had an *in vitro* degradation rate of 0.2 min^−1^ Lon_6_^−1^ (Fig. 2*B*), nearly one-sixth of the WT degron. Unsurprisingly, the H286C and H286S mutants exhibited low to mild *in vitro* degradation rates of 0.3 min^−1^ Lon_6_^−1^ and 0.5 min^−1^ Lon_6_^−1^, respectively. Notably, the H286R mutant had the slowest degradation rate among all examined substrates but was statistically indistinguishable from the H286D mutant (Fig. 2*B*). Collectively, the *in vitro* proteolysis data are consistent with the *in vivo* stability data, confirming that nature has selected histidine as the most favored C-terminal residue for recognition by Lon in the context of this degron group.

### Degron affinity (*K*_*D*_) measurements correlate well with degradation rates (*K*_*DEG*_)

We interpreted the observed differences in the degradation rates of the GFP-degrons as indicators of the relative affinity (*K*_*D*_) of Lon for the various degron sequences, all of which have a terminal histidine residue (Figs. 1 and 2). To further validate this inference and determine how degron affinity contributes to the overall degradation rate, we used the microscale thermophoresis (MST) technology to analyze the in-solution binding affinities between Lon and the GFP-appended degron variants.

**Figure 3:**
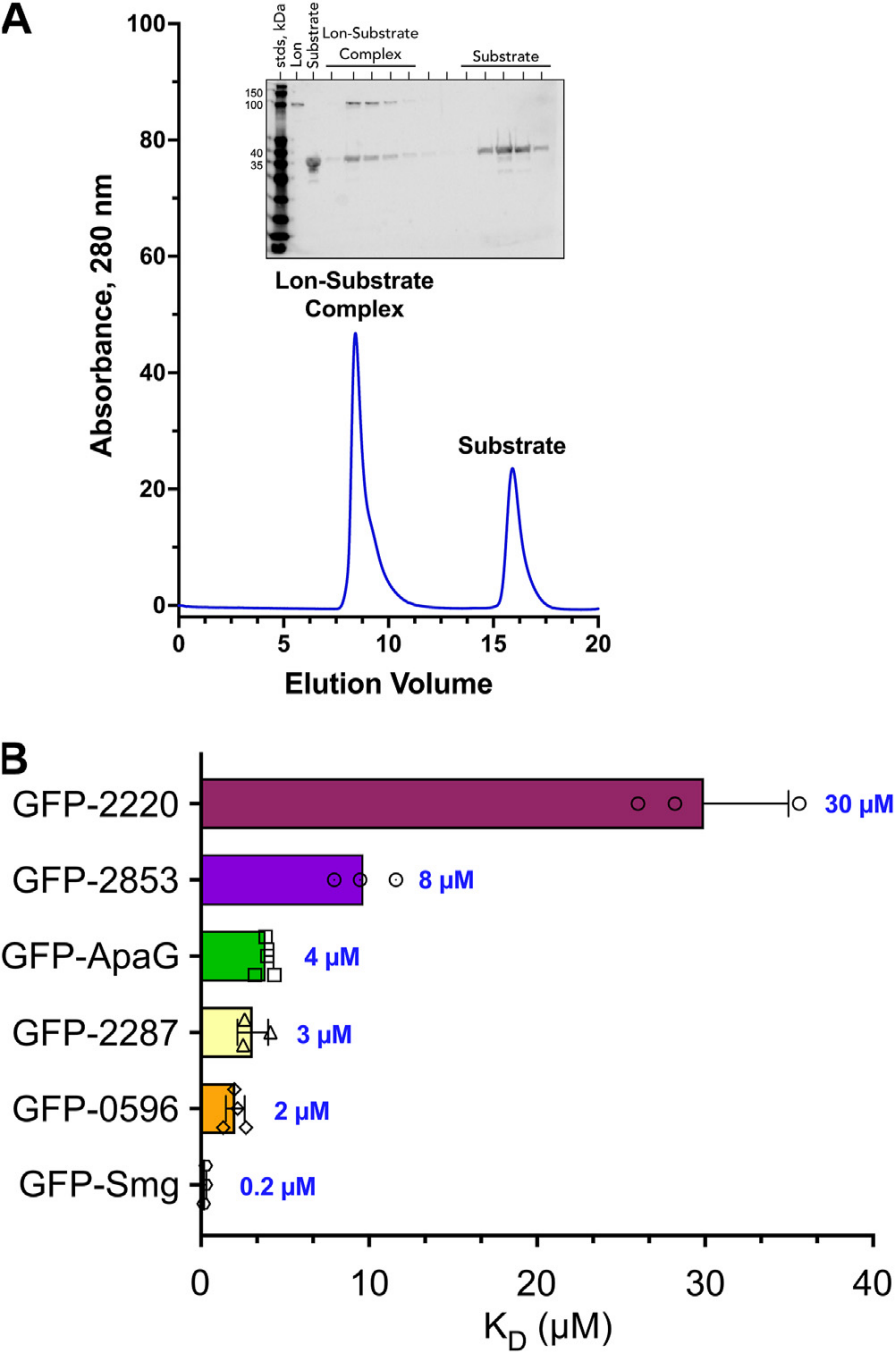
**Complex formation and binding affinity (*K*_*D*_) determination for Lon-GFP-degron variants.***A,* biochemical characterization of substrate-bound Lon complex. The Lon^E424Q/K722A^ was used for used for binding studies. Size-exclusion chromatography (SEC) traces showing substrate-bound Lon complexes eluting around 9 ml, an elution profile identical Lon^E424Q^ and Lon^WT^ (2). *B,* the substrate with the largest *K*_*D*_ (GFP-2220 at 29.93 μM) is also the slowest to be degraded per the *in vitro* degradation assays. Conversely, the substrate with the smallest *K*_*D*_ (GFP-smg at 0.2 μM) is the fastest to be degraded *in vitro*. The *K*_*D*_ values of the corresponding HD mutants are all too high (>50 μM) to be detected under our assay conditions by MST. Data presented in graphs are from three or more independent experiments (mean ± SD). MST, microscale thermophoresis.

The initial substrate recognition function of Lon is attributed to its N-terminal domain. Therefore, we used an ATPase-deficient and proteolytically inactive Lon mutant (Lon^E424Q/K722A^) in our MST binding assays to ensure that we are strictly evaluating the initial Lon–degron interaction step, without interference from the subsequent ATP-driven unfolding and peptidase activities of the hexamer. We first established that the Lon^E424Q/K722A^ mutant does indeed bind the reporter proteins using a Lon–substrate complex formation assay. In this assay, the GFP-smg reporter was incubated with Lon^E424Q/K722A^ in the presence of ATP and formation of the Lon–substrate complex was monitored by comigration of the smaller substrate with the larger Lon hexamer by size-exclusion chromatography. The size-exclusion chromatography analysis confirmed that the Lon^E424Q/K722A^ mutant, similar to the Lon^E424Q^ mutant (2, 46), was competent in substrate binding and formed a complex with the GFP-smg reporter (Fig. 3*A*). MST analysis of GFP reporter constructs carrying the unaltered WT degrons exhibited binding affinities (*K*_*D*_ values) ranging from 0.2 μM to 30 μM (Fig. 3 and Table 2). It should be noted that the measured *K*_*D*_ values correlate well with the degradation rates obtained from the proteolysis assays (Fig. 1). For example, GFP-smg has the fastest degradation rate and the highest affinity for yp-Lon. Notably, the *K*_*D*_ values for the H286D variants of these degrons were too large (>50 μM) to be measured accurately by MST under our assay conditions (Table 2). Nonetheless, changing the terminal histidine to aspartate has a dramatic negative effect on the binding affinity and recognition of this degron by yp-Lon.

Our data show that the smg degron has high affinity for Lon (Fig. 3 and Table 2). We have preciously shown that the Y2853 degron, the founding member of this group of degrons in our Lon^TRAP^ substrates, served as an autonomous Lon recognition degron, both when appended to a reporter protein and in the context of the native Y2853 protein (2). To examine whether the autonomous smg degron also targets the native Smg protein for proteolysis by Lon, we cloned, expressed, and purified the native Smg protein and assessed its propensity to serve as a Lon substrate. This analysis showed that Lon recognizes and degrades the native Smg protein efficiently (Fig. S2), with *K*_*DEG*_ = 1.9 min^−1^ Lon_6_^−1^.

### The predictive power of the C-terminal degron leads to identification of new Lon substrates

**Figure 4:**
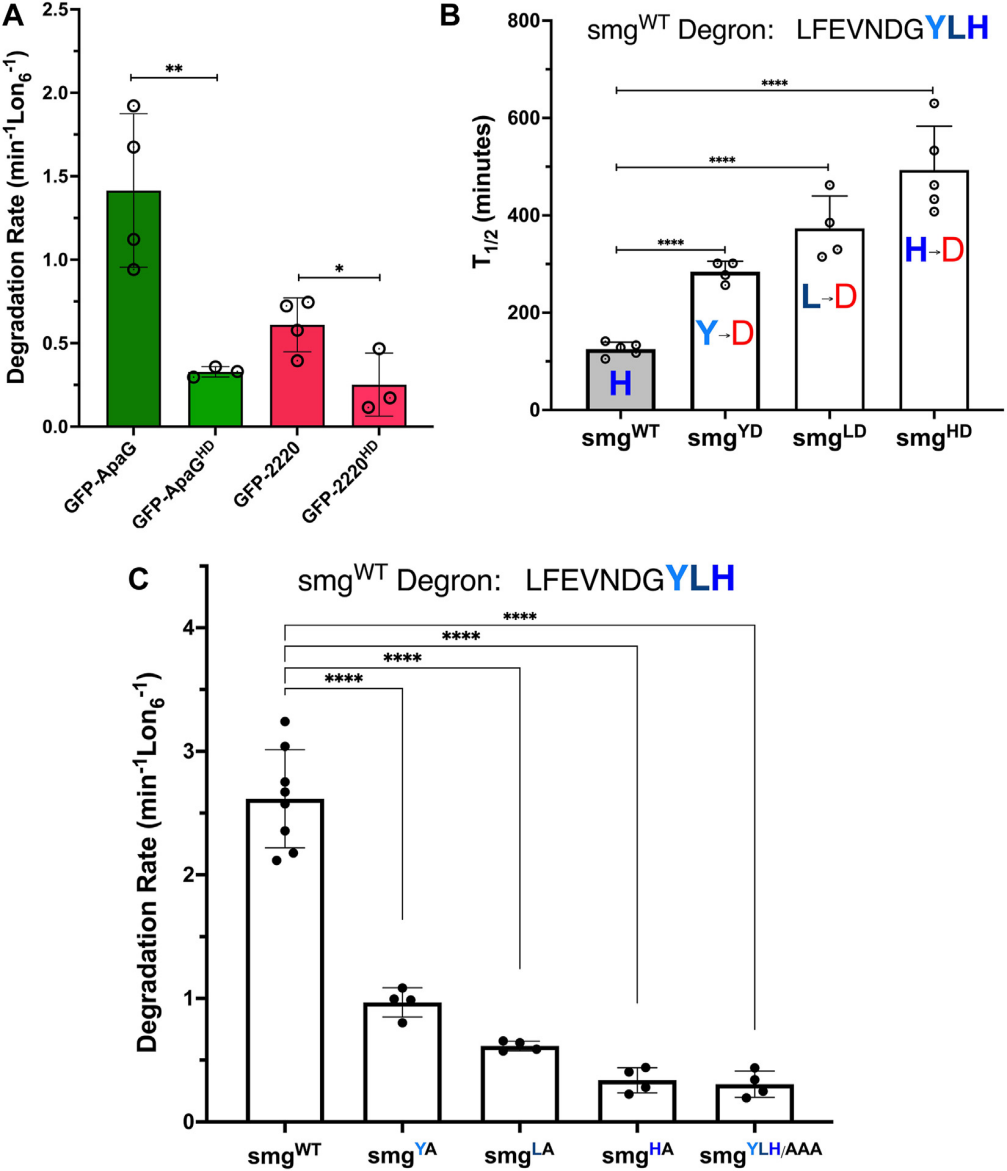
**Predicted Lon substrates exhibit a loss of degradation after mutation of the C-terminal residues.***A, Yersinia pestis* proteins y2220 and ApaG are predicted to be Lon substrates based on their C-terminal sequences (-PALIH and -DLSLH, respectively). Degradation rates of degron-tagged GFP and HD mutants were calculated and plotted to demonstrate the importance of the C-terminal histidine residue. *p* values from one-tailed *t* test between WT and “HD” degrons were calculated as ∗∗*p* = 0.0029 (2220), and ∗*p* = 0.0101 (ApaG). *B,* the C-terminal ultimate, penultimate, and antepenultimate residues of the GFP-smg degron were mutated to aspartate and their contributions to degradation by Lon was examined *in vivo. C, in vitro* analysis of the effect of Ala substitutions of the three terminal residues of the smg degron on recognition and degradation by Lon, showing that in addition to the terminal histidine residue, the penultimate leucine and antepenultimate tyrosine residues also contribute to recognition by Lon. *p* values from one-way ANOVA were both <0001 (∗∗∗∗). Data presented in graphs are from three or more independent experiments (mean ± SD).

Since all examined variants of this group of C-terminal histidine containing degron served as autonomous Lon recognition sequences and were degraded by Lon, both *in vitro* and *in vivo,* albeit at different rates, we hypothesized that additional Lon substrates containing variants of this degron must exist in bacterial proteomes. To test this hypothesis, we searched the yp (CO92) genome for encoded proteins that contain similar C-terminal degron motifs, more precisely proteins ending with the x–L/I–L/I/V–H–like sequences. Of the 3885 ORFs in the yp genome, 151 encode proteins ending in similar sequences, ∼3.9% of all the ORFs. For predictive testing of this hypothesis, we selected two proteins bearing variants of the putative degron sequences, ApaG (YPO0491) which ends with -PALIH motif and Y2220 (YPO2220) which ends with -DLSLH motif. According to our prediction, Lon should exhibit higher affinity for the ApaG C-terminal motif and recognize and degrade it faster than the weaker Y2220 C-terminal motif, which carries the disfavored aspartic acid (D) and serine (S) residues (2, 24, 47) (Fig. 4 and Table 1).

We appended the corresponding 20 terminal residues of the putative of ApaG and Y2220 degron sequences to the C terminus of GFP and expressed and purified each GFP-degron reporter protein. We also generated the HD mutants of both degrons to examine the effect of the terminal histidine residue on degron recognition by Lon. Analysis of the purified proteins in the *in vitro* proteolysis assays revealed that the predicted ApaG C-terminal sequence does indeed serve as an autonomous Lon recognition degron with a moderate *K*_*DEG*_ of 1.4 min^−1^ Lon_6_^−1^ (Fig. 4), comparable to two Lon^TRAP^ substates, GFP-0596 and GFP-2853 (Fig. 1 and Table 2). Conversely, the Y2220 C-terminal sequence, while still recognized and degraded by Lon, exhibited a much slower degradation rate, with a *K*_*DEG*_ of 0.6 min^−1^ Lon_6_^−1^ (Fig. 4*A*). While the degradation rates of the GFP-ApaG and GFP-2220 reporters by yp-Lon differed substantially, the terminal histidine residues of each degron still served as a key determinant for Lon recognition, as the HD mutants of these degrons exhibited significantly slower degradation rates with *K*_*DEG*_ values of 0.3 min^−1^ Lon_6_^−1^ and 0.2 min^−1^ Lon_6_^−1^, respectively (Fig. 4*A* and Table 2).

These GFP reporter constructs exhibited the same pattern of degradation as other members of this degron group, in which the H-ending WT constructs were degraded faster than the HD mutants. Furthermore, binding affinity measurements of the GFP-2220 and GFP-ApaG reporter constructs, using MST analyses, revealed that their Lon binding affinities (*K*_*D*_ values) correlated well with their *in vitro* degradation rates (*K*_*DEG*_). Interestingly, the GFP-2220 reporter, which had the slowest degradation rate of all GFP-degron reporters examined, had the lowest affinity (*K*_*D*_ = 30 μM) for Lon (Fig. 4*A*, and Table 2). GFP-ApaG had a comparatively moderate degradation rate (*K*_*DEG*_ = 1.4 min^−1^ Lon_6_^−1^) and a moderate binding affinity (*K*_*D*_ = 3.4 μM). Taken together, these data demonstrate the predictive power of this degron sequence in identifying new Lon substrates.

The *in vivo* and *in vitro* biochemical studies of all variants of this degron group highlight the key role of the terminal histidine residue for recognition by yp-Lon. However, there were clear indications throughout these investigations that additional residues, beside the terminal histidine, contribute to the affinity and proteolysis by Lon. To verify this conclusion, we mutated the ultimate (H), penultimate (L), and antepenultimate (Y) residues of the GF-smg reporter, carrying the high-affinity smg degron, to aspartate, and assessed their effect on recognition and proteolysis by Lon using the *in vivo* stability assay. This *in vivo* half-life analysis showed that all three C-terminal residues contribute to degradation by Lon, with the terminal histidine residue making the largest contribution to recognition by Lon (Fig. 4*B*). To further verify these results, we mutated the terminal (H), penultimate (L), and antepenultimate (Y) residues of the GF-smg reporter individually to alanine, along with a triple alanine substitution mutant (YHL/AAA) where all three terminal residues were changed to alanine. We expressed and purified all four mutants and evaluated their individual effects on recognition and proteolysis by Lon in the *in vitro* degradation assay.

This analysis confirmed that indeed substitution of the antepenultimate (GF-smg^YA^) and penultimate (GF-smg^LA^) residues of the degron to alanine have a significant impact on their recognition and degradation by yp-Lon, reducing the degradation rate (*K*_*DEG*_) from 2.6 min^−1^ Lon_6_^−1^ to 0.97 min^−1^ Lon_6_^−1^ and 0.61 min^−1^ Lon_6_^−1^, respectively (Fig. 4*C*). Expectedly, substitution of the terminal histidine residues to alanine (GF-smg^HA^) had a significantly more dramatic effect of the *in vitro* degradation of this reporter by yp-Lon, reducing the degradation rate to *K*_*DEG*_ = 0.3 min^−1^ Lon_6_^−1^ (Fig. 4*C*, Table 2). These data are consistent with the conclusion that additional residues of this C-terminal degron group, alongside the in variant terminal histidine, contribute to recognition and degradation by yp-Lon.

### Cleavage-site preferences of AAA+ Lon proteases

**Figure 5:**
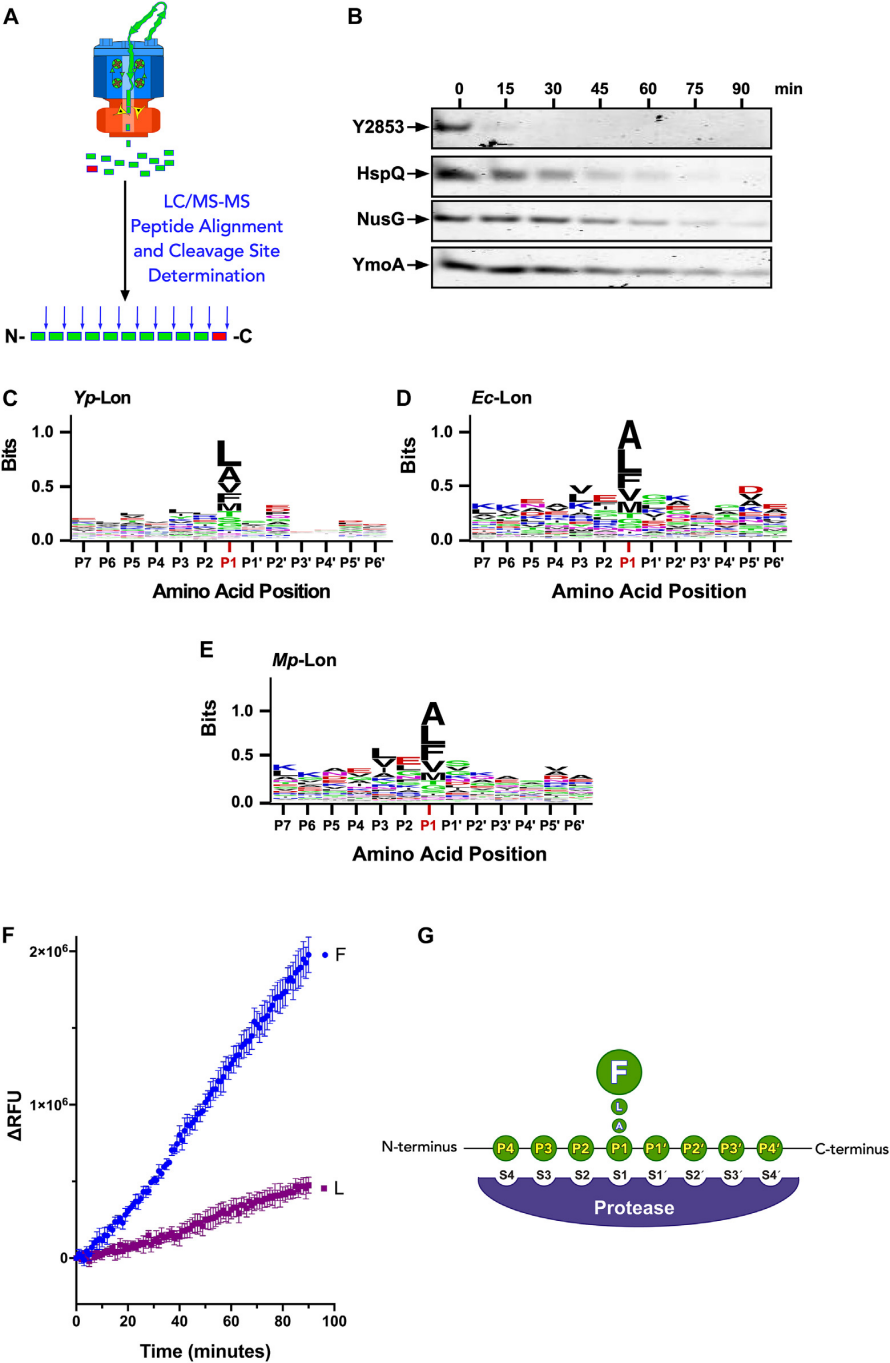
***In vitro* proteolysis of *Yersinia*-Lon substrates to identify its cleavage-site preferences.***A,* schematic representation of the proteolysis, LC-MS/MS analysis, and peptide cleavage-site determination. *B,* degradation assays were carried out at 37 °C in Lon activity buffer with 200 nM *Yp*-Lon_6_, 10 μM each substrate, and an ATP regeneration system. Aliquots were taken at designated time points, quenched with SDS-sample buffer, resolved by electrophoresis on 15% Tris-tricine gel and stained with Coomassie Brilliant BlueR250. The gel show is representative of two replicates. *C-F,* the peptides generated from digestion of each substrate by Lon protease were identified by LC-MS/MS, aligned, and their cleavage sites determined. Sequences of six amino acids on either of the cleavage sites were analyzed by Weblogo to derive the cleavage-site preferences of (*C*) *Yp*-Lon, (*D*) *Ec*-Lon, and (*E*) *Mp*-Lon. *F,* fluorogenic peptide cleavage assay was used to determine the P1-site preferences of Lon protease, graphically illustrated in panel (*G*), illustrating Lon preference for phenylalanine residues at the P1 site. Yp, *Yersinia pestis;* Ec-Lon, *Escherichia coli* Lon; Mp, *Mycoplasma pneumoniae.*

Substrate recognition by the N-terminal domain of Lon is followed by substrate engagement with the central ATPase domain, which triggers ATP-dependent unfolding and translocation of the polypeptide to the peptidase chamber wherein it is dissected to smaller peptides. Surprisingly, little is known about the cleavage-site preferences of Lon or the range of peptides generated by its peptidase domain. With the availability of multiple native known and novel Lon substrates (2), we investigated the cleavage-site preferences of Lon protease. We cloned the yp *y2853, hspQ, nusG, fur, y0390, rsuA,* and *crp* genes, expressed, and purified the protein products (Fig. S3). We subjected the individual purified proteins to *in vitro* proteolysis by yp-Lon. As expected, the native protein substrates exhibited a range of degradation rates. For instance, Y2853, a native Lon substrate, the C-terminal sequence of which we characterized as an autonomous Lon recognition degron (Fig. 1), was degraded to completion within 15 min, whereas a small percent (10%) of CRP remained intact after 90 min incubation with Lon (Fig. 5*A* and (2)). The observed difference in degradation rates of these substrates is likely due to a combination of the variable affinity of Lon (*K*_*D*_) for the respective degron sequences and the intrinsic stability of each protein. We utilized this information to adjust the degradation endpoint of each substrate to ensure each protein was degraded to completion, such that little to no intact protein was detected by Coomassie Brilliant Blue staining of the reaction products. We trichloroacetic acid precipitated the proteolyzed components of the assay to separate the soluble digested peptides from the intact Lon protease and creatine kinase (a component of the ATP regeneration system). To determine the cleavage-site preferences and distribution of peptide products, we subjected the acid soluble peptide products to analysis by LC-MS/MS (Fig. 5*B*).

We identified the peptide products of these yp*-*Lon protease substrates by mass spectrometry to determine Lon cleavage-site preferences (Fig. 5). Each cleaved peptide product, except for the N- and C-terminal fragments, is the product of two cleavage events. The C-terminal residue of each peptide corresponds to the P1 residue or amino acid residues after which the Lon peptidase domain cleaves the unfolded polypeptide, whereas the N-terminal residue of the same peptide corresponds to the P1′ residue or amino acid residues before which the Lon peptidase domain cleaves the polypeptide. Weighted matrix analysis of all the peptide products for each substrate, using Weblogo, show 13 residues centered on the P1 residue and flanked by six residues on either side. Amino acids at the P1 position showed a high degree of conservation, with leucine and alanine being the most frequently observed amino acids, whereas residues at all other positions showed little or no conservation (Fig. 5*C*). Altogether, the analysis of the peptide products of these native yp-Lon substrates implied that the *Yersinia* Lon prefers to cleave translocated polypeptides after leucine, alanine, phenylalanine, and valine residues.

### Cleavage-site preferences of Lon proteases are conserved

Previous studies from our lab revealed similarities in substrate specificities of Lon proteases from evolutionarily distant bacterial species, *E. coli* and MP (24). We hypothesized that similarities between Lon proteases from these diverse bacterial species may also be extended to their cleavage-sites preferences. To test this hypothesis, we utilized MP-Lon protease and a known substrate, the λ-cI-N-ssrA_MP_, which we had previously characterized (24). Moreover, we purified two additional known Lon substrates, ribosomal proteins S2 and L9 (21) for cleavage-site analyses of MP and Ec-Lon proteases. We performed degradation assays of these substrates by either MP*-*Lon or Ec*-*Lon and analyzed the peptide products by LC-MS/MS. Consistent with our prediction, both Ec-Lon and *Mycoplasma* Lon proteases exhibited cleavage-site preferences that were similar to the *Yersinia* Lon protease, where leucine, alanine, and phenylalanine were among the most frequently identified P1-site residues (Fig. 5, *C*–*F*). The observed cleavage site conservation suggests high degree of similarity between the proteolytic active site architecture of Lon proteases from diverse bacterial species.

These observations implied that the peptidase domain of Lon has evolved to cleave preferentially after leucine and alanine residues, which occur at much higher frequency in protein sequences (48), with leucine ranked first and alanine ranked second among the 20 standard amino acids (Table S3). Curiously, our data also show that Lon prefers to cleave after phenylalanine residues. Therefore, if one considers amino acid occurrence frequencies, the preference of Lon to cleave after phenylalanine is more striking, as it occurs comparatively infrequently in protein sequence and is ranked 14th among the 20 canonical amino acid residues (Table S3). To further verify Lon cleavage-site preferences, we examined the cleavage of short fluorogenic peptide by Lon. To ensure that the observed peptide cleavage was governed solely by the peptidase domain of Lon, we used a well-characterized ATPase-deficient Walker B mutant of yp-Lon (Lon^E424Q^), which is still capable of peptidase activity (2, 46). Based on highly abundant cleavage-site sequences in our proteomic analysis, we designed short pentapeptides Tyr^1^-Glu^2^-X^3^-Gly^4^-Lys^5^ (YEXGK), where position 3 corresponds to the P1 site and was changed to either Leu or Phe residues (Fig. 5). Each peptide contained a 3-nitrotyrosine quencher at position 1 and a modified lysine residue at position 5 bearing a side chain linked 2-aminobenozyl fluorophore (Lys-Abz). Proteolytic cleavage of each peptide by yp-Lon, anywhere between the two terminal residues, should release the Lys-Abz fluorophore and produce a quantifiable increase in the fluorescence signal. As expected, the yp-Lon^E424Q^ variant rapidly cleaved the 3-nitrotyrosine-Glu^2^-Leu^3^-Gly^4^-(Lys^5^-Abz) reporter peptide (Fig. 5*G*). Most interestingly, replacement of Leu with Phe resulted in a substantial enhancement of peptide cleavage rate, suggesting that Lon does indeed prefer cleavage after Phe residues.

### Cleavage by Lon proteases yield short peptides ranging from 8 to 35 amino acids in length

**Figure 6:**
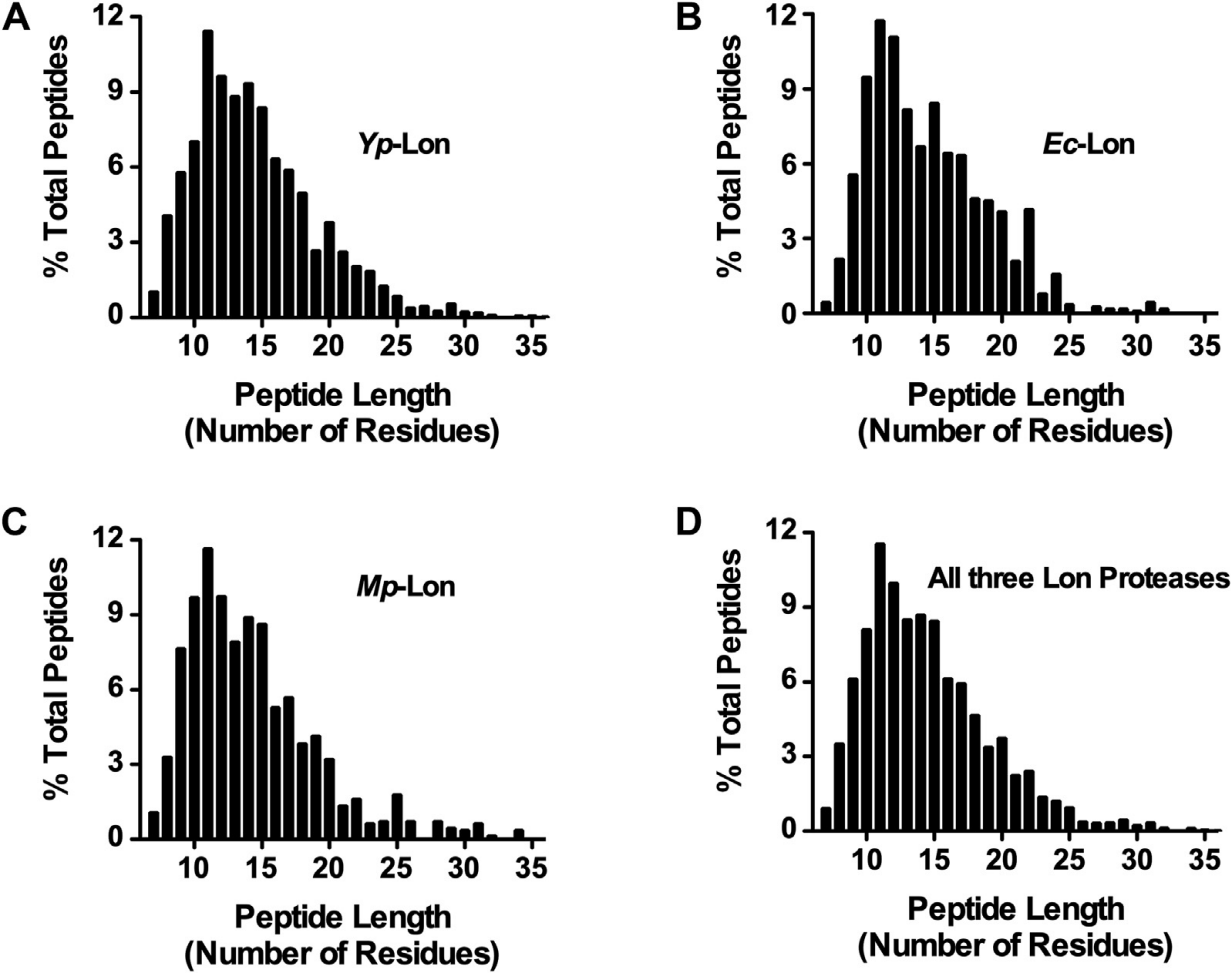
**Peptide length distribution of Lon degradation products.***A, Yp*-Lon, (*B*) *Ec*-Lon, (*C*) Mp-Lon, and (*D*) all three Lon proteases. Mass spectrometry identified peptides ranging from a length of 7 to 35 amino acid residues. The distribution of the peptide lengths was analyzed by calculating the percentage of the peptide of a certain length in the pool all peptide products. Yp, *Yersinia pestis;* Ec-Lon, *Escherichia coli* Lon; Mp, *Mycoplasma pneumoniae.*

The analysis of the preferred cleavage sites by Lon protease led us to postulate that after the unfolded substrate is translocated through the central pore and reaches the protease chamber, the proteolytic active site scans the bound polypeptide and cleaves after detection of its preferred residues. To determine the range and distribution of peptides produced by Lon, we measured the length of the peptides generated by all three Lon proteases. We found that the yp Lon generated peptides ranging from 7 to 35 amino acid residues in length, where average length of the peptides was 11 residues and majority of the peptides were 9 to 20 amino acid residues in length (Fig. 6*A*). Interestingly, the range of peptide products generated by the Ec-Lon and MP-Lon proteases were indistinguishable from the *Yersinia* Lon protease (Fig. 6, *B*–*D*).

## Discussion

AAA+ proteases play a pivotal role in protein homeostasis. As a widely distributed and highly conserved member of the family, Lon protease participates in a diverse array of cellular processes by regulating the intracellular levels of a growing number of key regulatory proteins. Lon encounters a diverse set of protein substrates during normal growth and under various physiological stress conditions. One class of Lon substrates constitute unfolded or damaged proteins with exposed hydrophobic sequence elements that are recognized by Lon. In addition to this wide selection of damaged or misfolded proteins, Lon also recognizes a growing number of natively folded regulatory proteins. Members of this class of substrates must carry Lon recognition degrons, located at the amino, carboxy, or internal segments of the target proteins that become conditionally available for recognition by Lon. Degradation of these natively folded proteins often involves exquisitely fine-tuned and spatiotemporal controlled recognition and degradation, often aided by adaptor proteins (2, 49). Our working hypothesis is that there must exist several classes of sequence elements (degrons) that signal recognition of natively folded substrates by Lon. Although the Lon recognition elements of most Lon substrates have not been deciphered, this postulate is consistent with previous studies of Lon substrates carrying N-terminal, internal, or C-terminal recognition elements (2, 24, 31, 36, 37, 39). However, widely distributed Lon recognition degrons which are broadly present in numerous known and new Lon substrates, in diverse bacterial species, has not been identified.

Our exploration of Lon substrate recognition rules uncovered a class of C-terminal yp-Lon degrons, variants of which are found in numerous known and new Lon substrates and widely distributed in numerous bacterial species. A key sequence hallmark of this class of degrons is an invariant C-terminal His residue, preceded by two hydrophobic residues (x–L/I–L/I/V–H), which tolerate notable sequence variation. Our findings also demonstrate the autonomous nature of this degron, in that appending a variant of this degron group to the C terminus of a nonsubstrate reporter protein, GFP, results in degron-dependent recognition and degradation. The terminal His residue serves as a critical Lon recognition determinant, as its substitution to any of the other 19 amino acid residues results in substantial reduction in substrate affinity (*K*_*D*_) and consequently efficient recognition and degradation by Lon (Fig. 2). It is well established that bacterial proteases disfavor acidic terminal residues (2, 24, 43, 45). Our data demonstrate that substitution of the terminal His residue to Asp has a dramatic effect on recognition by Lon, both *in vivo* and *in vitro*. Intriguingly, we find that Lon also disfavors terminal basic (Arg and Lys) residues (Fig. 2). Given that bacterial proteases disfavor acidic terminal residues (45), we speculate that this aversion to basic terminal residues might be shared by other bacterial proteases. Consistent with this notion, a recent study demonstrated the strong effect of basic terminal residues on protein expression and *in vivo* stability (50). Future studies are required to determine whether this aversion to terminal basic residues (Arg and Lys) also extends to other bacterial proteases.

Most significantly, knowledge of the existence of this widely distributed degron group affords the predictive power to identify new Lon substrates in bacterial proteomes. We identified over 150 potential Lon substrates in the annotated yp proteome, totaling to up to 4% of the predicted 3885 encoded proteins. Analysis of two of predicted Lon substrate (ApaG and Y2220) from this group confirmed the predictive power of this approach and demonstrated that their C-terminal sequences do indeed serve as autonomous Lon degrons (Fig. 3). A limited analysis of gram-negative and gram-positive bacterial species of interest revealed that they contain a sizeable number of proteins harboring variants of this widely distributed C-terminal degron group. For instance, approximately 180 proteins in *E. coli* (∼4.3% of ORFs), 30 proteins in *Francisella tularensis* (∼1.7% of ORFs), 110 in proteins *Bacillus subtilis* (∼2.6% of ORFs), 186 proteins in *Klebsiella pneumoniae* (9.5% of ORFs), 14 proteins in *Mycoplasma* genitalium (∼3% of ORF), and 90 proteins in *Caulobacter crescentus* (∼2.3% of ORFs) carry variants of this degron group. Consistent with this conclusion, previously known Lon substrates SulA in *E.* coli (38, 40), Y2853 in yp (2), and two recently characterized *C. crescentus* proteins, LarA and SciP, which carry variants of this degron, were shown to be degraded by Lon (51, 52). Therefore, it is highly likely that many of these predicted substrates are recognized and degraded by their respective Lon proteases under defined physiological conditions.

We and others have shown that Lon recognizes multiple sequence elements in its known degrons (2, 24, 31, 37, 39). Consistent with these studies, our analysis of the penultimate and antepenultimate residues of this class of this class of C-terminal degrons clearly demonstrates that additional sequence signals of this degron are important for efficient recognition by Lon, as their substitution to Asp or Ala renders tagged substrates more resistant to proteolysis (Fig. 4). Therefore, we conclude that multiple sequence elements of this degron group contribute to recognition and degradation by Lon protease.

Numerous proteases exhibit defined specificity for cleaving after preferred amino acids around the scissile bond. To gain a deeper and more nuanced understanding of the cleavage-site preferences of Lon protease, we used Lon proteases from three bacterial species (yp, *E. coli*, and MP) and 17 known and new native substrates. Our mass spectrometry analysis showed that a large number of peptides generated by all three Lon proteases terminate with Leu and Ala residues, with Phe appearing as the next most frequently observed P1 site residue (Fig. 5). Since Leu occurs at much higher frequency than Phe in all proteins, and in the Lon substrates we examined (Table S3 and (48)), we employed a fluorogenic peptide cleavage assay to directly evaluate the P1-site preferences of Lon protease for peptides carrying either a central Leu or Phe residue. Indeed, this analysis revealed that the peptidase domain of Lon displays a prevailing preference for Phe over Leu as the P1 site residue (Fig. 5*G*). Previous cleavage site preference studies of the *E. coli* Lon protease with individual substrates (53, 54, 55, 56) concluded that Lon prefers cleaving after Leu and Ala residues. Since Leu and Ala occur at much higher frequencies than Phe in all proteins (Table S3 and (48)), we reasoned that perhaps this disparity in the frequency of occurrence might have masked the identification of the primary cleavage site preference of the Lon peptidase sites. For instance, a previously analyzed Lon substrate, SulA, has 21 Leu but only 2 Phe residues (54). Therefore, it is highly likely that peptides ending with a secondarily preferred Leu residue would appear most frequently in the cleaved peptide products of Lon. Similarly, analysis of ribosomal protein S2 (55) showed that a preponderance of the major cleavage products (33%) were cleaved after Phe, whereas peptides ending with Leu constitute only 17% of the major cleavage products, despite having 21 Leu and 12 Phe residues (Fig. 3 in reference (54)). Therefore, based on the sum of these data, we conclude that Lon has a primary preference for cleaving after Phe residues, in the absence of which it cleaves after Leu and Ala residues. Since protease cleavage-site preferences are determined by the structural arrangements of their respective active sites, high-resolution structural information is required to better understand how the peptidase active sites of Lon protease position translocated polypeptides for hydrolysis.

Our mass spectrometry analysis also showed that all three proteases generate peptides of 7 to 35 amino acid residue in length, with a preponderance of 10 to 11 residues peptides (Fig. 6). Remarkably, we observed no obvious difference in cleavage-site preferences for substrate carrying C-terminal, N-terminal, or internal degrons, suggesting that the orientation of the substrate in the peptidase active site is not determined by the direction of translocation into the proteolytic chamber. Furthermore, the cleavage pattern of Y2853, a rapidly degraded native substrate, is indistinguishable from Crp, a native substrate with a much slower degradation rate (Fig. S3), suggesting that substrate degradation rate does not impact the cleavage pattern or cleavage-site preferences of Lon. This conclusion is in agreement with the finding that cleavage-sites for Lon are independent of ATP hydrolysis and translocation rates (56). The high degree of cross-species conservation of Lon protease function suggests that the substrate recognition and cleavage-site preferences are likely to be widely conserved in eubacteria.

## Experimental procedures

### Protein purification

*E. coli* BL21 (DE3) cells harboring the plasmid pET28b-*YP*-Lon were grown in LB broth at 37 °C to OD_600_ of 0.5 to 0.7. The expression of *Yersinia* Lon proteases was induced by the addition of IPTG to a final concentration of 1 mM. Cells were grown for an additional 16 h at 16 °C, harvested by centrifugation at 3700*g* for 1 h, and stored at −80 °C. Cell pellets containing *Yersinia* Lon protease were resuspended in cold buffer A (50 mM KHPO_4_ pH 6.9, 10% glycerol, and 1 mM DTT) at 4 °C, lysed by sonication on ice, and the cell lysate was subjected to centrifugation at 10,000*g* for 30 min to remove cell debris. The cleared cell lysate was passed through buffer A equilibrated P11-cellulose resin by gravity-flow and the column was washed extensively with buffer A to remove unbound proteins. The bound *Yersinia* Lon was eluted in 10 ml of buffer B (250 mM KHPO_4_ pH 6.9, 10% glycerol, and 1 mM DTT). The resulted protein was buffer exchanged into buffer C (20 mM KHPO_4_ pH 6.9, 50 mM KCl, 10% glycerol, and 1 mM DTT), applied onto a Mono-Q HR 10/10 column, washed with 20 CV of buffer C, and the bound protein was eluted by the application of a linear KCl gradient from 0% buffer C to 100% buffer D (20 mM KHPO_4_ pH 6.9, 1 M KCl, 10% glycerol, and 1 mM DTT). Fractions containing *Yersinia* Lon protease were pooled, concentrated, and stored at −80 °C. The expression and purification procedure for *E. coli* Lon and *Mycoplasma* Lon is as described (24).

*E. coli* strain BL21 (DE3) harboring the plasmids expressing each of the potential *Yersinia* Lon native substrate was grown in 6 L of LB broth at 37 °C to *A*_600_ of 0.7. The expression of the target protein was induced by the addition of IPTG to a final concentration of 1 mM. Cells were grown for an additional 3 h at 37 °C, harvested by centrifugation at 3700×*g* for 1 h, and stored at −80 °C. Cell pellets containing each of the potential *Yersinia* Lon substrates were resuspended in buffer E (50 mM KHPO_4_ pH6.9, 500 mM KCl, 1 mM EDTA, and 2 mM beta-mercaptoethanol [β-ME]), lysed by sonication on ice, and subjected to centrifugation at 10,000×*g* for 30 min to remove cell debris. The cleared cell lysates were mixed with 2 ml nickel-nitrilotriacetic acid (Ni-NTA) slurry (sigma) pre-equilibrated in buffer E and incubated at 4 °C for 2 h to allow binding of the targeted protein to the resin. The resin was washed with Buffer A and the bound proteins were eluted in 25 ml of Buffer F (50 mM KHPO_4_ pH6.9, 500 mM KCl, 1 mM EDTA, 2 mM β-ME, and 200 mM imidazole). The eluted protein was concentrated, and buffer was changed into buffer C, and further purified on either a Mono-Q HR 10/10 column (GE healthcare) or Mono-S HR 10/10 column (GE healthcare), depending on the pI of the protein being purified, as described above, Fractions containing pure target protein were pooled, concentrated, and stored at −80 °C for future use.

For the overexpression of *E. coli* S2 and L9, the clone containing the expression construct of the target protein was selected from the ASKA library (57) and grown in 6 L of LB broth at 37 °C to *A*_600_ of 0.7. The expression of the target protein was induced by the addition of IPTG to a final concentration of 1 mM. Cells were grown for an additional 16 h at 16 °C, harvested by centrifugation at 3700×*g* for 1 h, and stored at −80 °C. Cell pellets containing either S2 or L9 were resuspended in buffer G (50 mM KHPO_4_ pH6.9, 500 mM KCl, 1 mM EDTA, 4 M urea, and 2 mM β-ME) lysed by sonication on ice, and the crudes cell lysates were subjected to centrifugation at 10,000×*g* for 30 min to remove cell debris. The cleared cell lysates were mixed with 2 ml Ni-NTA slurry (sigma) pre-equilibrated in buffer G and incubated at 4 °C for 2 h to allow binding of the targeted protein to the resin. The resin was then washed with buffer G and the bound proteins were eluted in 25 ml of buffer H (50 mM KHPO_4_ pH6.9, 500 mM KCl, 1 mM EDTA, 4 M urea, 2 mM β-ME, and 200 mM imidazole). The eluted protein was concentrated, buffer exchanged into buffer C, and applied onto a Mono-Q HR 10/10 column (GE healthcare). The column was washed with 20 CV of buffer C, and the bound protein was eluted by the application of a linear KCl gradient from 0% buffer C to 100% buffer D. Fractions containing pure target protein were pooled, concentrated, aliquoted, and stored at −80 °C.

For expression of all GFP-degron reporters, *E. coli* W3110 (DE3) cells harboring variants of the plasmid 2BT-His_6_-CP6, were grown in LB broth at 37 °C to *A*_600_ of 0.5 to 0.7. At this point, expression of the variant was induced by addition of IPTG to a final concentration of 1 mM. Cells were grown for an additional 3 h at 37 °C, harvested by centrifugation at 3300*g*, and the resulting cell pellets were stored at −80 °C. Frozen cell pellets were resuspended in 20 ml lysis buffer (50 mM Tris–HCl pH 8, 1 M KCl, 10 mM imidazole, 2% glycerol, and 1 mM DTT) per liter culture pellet and lysed by sonication at 4 °C. The cleared lysate was incubated with equilibrated Ni-NTA beads for 1 h, washed extensively with lysis buffer to remove unbound proteins, and the His_6_-tagged protein was eluted in buffer I (50 mM Tris–HCl pH 8, 50 mM KCl, 125 mM imidazole, 2% glycerol, and 1 mM DTT). Elution fractions were pooled, concentrated, and loaded onto Superdex 75 increase 10 to 300 Gl equilibrated in gel filtration buffer (50 mM Tris–HCl pH 8, 100 mM KCl, 5 mM MgCl2, 10% glycerol, and 1 mM DTT). Fractions containing pure target protein were pooled, concentrated, aliquoted, and stored at −80 °C.

### Biochemical assays

For Lon protease, SDS-PAGE based *in vitro* proteolysis assay was carried out in an 80 μl reaction mixture containing Lon activity buffer (50 mM Tris–HCl pH 8.0, 100 mM KCl, 10 mM MgCl_2_, 1 mM DTT, and 10% glycerol), ATP regeneration system (50 mM creatine phosphate, 80 μg/ml creatine kinase, and 4 mM ATP), 5 μM substrate, and Lon protease (either 100 mM MP*a* Lon_6_, or 200 nM yp*s* Lon_6_, or 200 nM *E. coli* Lon_6_). For ClpXP, degradation assays were performed at 30 °C in a buffer containing proteolysis buffer (25 mM Hepes-KOH (pH 7.6), 200 mM KCl, 5 mM MgCl_2_, 10% glycerol, and 0.032% NP-40) with an ATP regeneration system containing 4 mM ATP, 16 mM creatine phosphate, and 0.32 mg/ml creatine kinase. Reaction contained 100 nM ClpX_6_, 300 nM ClpP_14_, and each substrate at indicated concentration.

Fluorescent *in vitro* proteolysis assays were carried out at 37 °C in a 30 μl reaction mixture containing Lon activity buffer, 10 μM substrate, and 200 nM yp Lon_6_. Reactions were thermally equilibrated and loaded into a black 364-well flat-bottom plate (Corning). ATP regeneration system (50 mM creatine phosphate, 80 μg/ml creatine kinase, and 4 mM ATP) was added to initiate the reaction, and samples were monitored for fluorescent signal (485 excitation/525 emission) over time in either a SpectraMax iD3 or iD5 plate reader (Molecular Devices) with photomultiplier tubes (PMT) sensitivity set to low. The slope of the linear portion of the curve was used to calculate K_DEG_.

Fluorogenic peptide cleavage assays (60 μl) were carried out in 384-well black plates (Corning) at 37 °C using SpectraMax iD3 plate reader (320 excitation/420 emission). Reactions containing 1 μM Lon E424Q, 10 μM fluorogenic peptide (GenScript), and Lon activity buffer (50 mM Tris–HCl pH 8.0, 10 mM MgCl_2_, 1 mM DTT, and 10% glycerol) were incubated at 37 °C for 10 min and then the proteolysis was catalyzed by addition of 5 mM ATP. Control reactions were “catalyzed” with MiliQ water. Reactions were monitored for increases in their fluorescent signal over time with PMT sensitivity set to low. The signal from the control reactions was subtracted from the data to account for evaporation of the fluorophore. Cleavage rates were determined by measuring the gain in fluorescence in early linear time points.

For MST assays, Lon^E424Q/K722A^ was serially diluted with MST buffer (50 mM Tris–HCl pH 7.8, 150 mM NaCl, 10 mM MgCl_2_, 0.05% Tween-20) so that the highest concentration was 45.15 μM and the lowest concentration was 1.4 nM. Each sample was treated with 600 nM of fluorescent binding partner (GFP variants) for 5 min at room temperature. Then, samples were applied to capillary tubes and loaded into Monolith NT.115 Series (Nanotemper) for MST. ΔFnorm values were exported into GraphPad Prism (https://www.graphpad.com/features) for statistical analysis and generation of binding curve. For all biochemical assays, data is represented as the mean ± SD from at least three independent replicates.

### Mass spectrometry analysis

To prepare the peptide samples for identification of peptides, *in vitro* proteolysis reactions were set up and allowed sufficient time to reach completion. Upon completion of the reaction, 20 μl of 10 mg/ml bovine serum albumin was added to the reaction. Following this 30 μl of 100% trichloroacetic acid (w/v) was added to the reaction mixture. The mixture was incubated on ice for 30 min to allow the precipitation of intact proteins. The mixture was spun at 10,000×*g* for 15 min to separate the precipitated proteins. The resulting supernatant containing the peptide products was used for mass spectrometry analysis.

Samples were manually loaded onto a 3 cm 250 μm I.D. C18 column (C18 Phalanx). Column was attached in front of a 10 cm (100 μm I.D.) C18 column (C18 Phalanx) and an HPLC gradient was run on the Orbitrap. Peptides were fragmented (1+ and higher peptides chosen for tandem mass spectrometry). Data was analyzed using Inspect (https://proteomics3.ucsd.edu/ProteoSAFe/index.jsp) with no enzyme specified. Peptides with *p* values of <0.01 were used for analysis. Database was constructed with the number of sequences corresponding to the number of times a spectrum was identified from that position. http://weblogo.berkeley.edu/logo.cgi was used for analysis.

### *In vivo* protein stability assay

*E. coli* W3110 (DE3) cells, harboring a plasmid encoding a C-terminal variant of GFP-0596, were grown in LB broth at 37 °C to *A*_600_ of 0.5 to 0.7. Expression of the GFP-0596 variant was induced with addition of IPTG to a final concentration of 1 mM and grown at 37 °C for 30 min. Protein synthesis was inhibited by addition of chloramphenicol at a final concentration of 500 μg/ml and the cell culture was allowed to grow for another 20 min. One milliliter aliquots of the culture were harvested by centrifugation at 3300×*g* for 10 min. The supernatant was discarded, and the cells were resuspended in M9 minimal media with appropriate antibiotics to reduce the effects of autofluorescence from the media. One hundred microliters of cell suspension containing equal number of cells were pipetted into wells of a 96-well black clear bottom plate. *In vivo* stability of the construct was recorded by monitoring fluorescent signal (485 excitation/525 emission) for 3 h from the bottom of the plate in a SpectraMax iD3 plate reader. PMT sensitivity was set to medium, integration time was set to 400 ms, and orbital shaking was used to avoid cell settling. The natural log of the fluorescent signal was plotted against time, and the linear portion of the data was determined to be **k**, the degradation rate constant. The half-lives of each GFP-0596 variant were calculated using the equation t_1/2_ = ln (2)/k.

## Data availability

Any additional information necessary to analyze the data presented in this article is readily available from the corresponding author upon request.

## Supporting information

This article contains supporting information.

## Conflict of interest

The authors declare that they have no conflicts of interest with the contents of this article.

## Supporting information

Cleavage site peptides analysis
