# Supplementary material for: Substrate recognition and cleavage-site preferences of Lon protease
Source: J Biol Chem. 2025 Feb 27;301(4):108365. doi: 10.1016/j.jbc.2025.108365 (PMC11986505; doi:10.1016/j.jbc.2025.108365)
Supplement: Supplemental Tables [file mmc2.docx]

**Table S1.** Half-life (t_1/2_) values for the GFP-0596 terminal residue variants

| **Substrate** | **t_1/2_ ± SEM (minutes)** |
| --- | --- |
| **GFP-0596^H286^ (WT degron)** | 152 ± 5 |
| **GFP-0596^H286V^** | 283 ± 25 |
| **GFP-0596^H286P^** | 319 ± 26 |
| **GFP-0596^H286I^** | 368 ± 25 |
| **GFP-0596^H286M^** | 376 ± 35 |
| **GFP-0596^H286W^** | 384 ± 31 |
| **GFP-0596^H286L^** | 388 ± 21 |
| **GFP-0596^H286A^** | 389 ± 12 |
| **GFP-0596^H286F^** | 398 ± 21 |
| **GFP-0596^H286T^** | 398 ± 22 |
| **GFP-0596^H286N^** | 401 ± 45 |
| **GFP-0596^H286Q^** | 402 ± 36 |
| **GFP-0596^H286E^** | 425 ± 6 |
| **GFP-0596^H286G^** | 452 ± 32 |
| **GFP-0596^H286Y^** | 481 ± 53 |
| **GFP-0596^H286K^** | 498 ± 42 |
| **GFP-0596^H286D^** | 548 ± 44 |
| **GFP-0596^H286R^** | 619 ± 51 |

^(^**^a^**^)^Values calculated from at least 3 independent repeats.

**Table S2:** Substrate used for determining cleavage site preferences of Lon protease.

| **Protease** | **Substrates** |
| --- | --- |
| *Y. pestis* Lon | RsuA, Y2853, CRP, NusG, YmoA, Fur, HspQ, and Y0390 |
| *E. coli* Lon | λ-cI-N-ssrA_MP_, and Ribosomal Proteins S2 and L9 |
| *M. pneumonia* Lon | λ-cI-N-ssrA_MP_, S2, and L9 |

**Table S3:** Amino Acid Frequencies in Lon Substrates**.**

| **Amino Acid** | **AA Frequency**  **(Lon Substrates)** | **AA Frequency**  **(All Protein)(**[**46**](#_ENREF_46)**)** |
| --- | --- | --- |
| 1. **LEU** | **10.6** | **9.2** |
| 1. **GLU** | **8.4** | **6.4** |
| 1. **ASP** | **7.6** | **6.0** |
| 1. **VAL** | **7.1** | **7.1** |
| 1. **ARG** | **6.3** | **5.1** |
| 1. **ALA** | **6.2** | **8.3** |
| 1. **ILE** | **5.6** | **5.5** |
| 1. **LYS** | **5.4** | **5.4** |
| 1. **GLY** | **5.1** | **7.3** |
| 1. **SER** | **5.0** | **6.1** |
| 1. **PRO** | **5.0** | **4.8** |
| 1. **THR** | **4.9** | **5.6** |
| 1. **GLN** | **4.8** | **3.7** |
| 1. **TYR** | **3.8** | **3.6** |
| 1. **PHE** | **3.3** | **4.2** |
| 1. **MET** | **3.2** | **2.1** |
| 1. **HIS** | **3.1** | **2.5** |
| 1. **ASN** | **3.0** | **4.3** |
| 1. **TRP** | **0.9** | **1.5** |
| 1. **CYS** | **0.9** | **1.4** |
