# Supplementary material for: Substrate recognition and cleavage-site preferences of Lon protease
Source: J Biol Chem. 2025 Feb 27;301(4):108365. doi: 10.1016/j.jbc.2025.108365 (PMC11986505; doi:10.1016/j.jbc.2025.108365)
Supplement: Supplementary Figures [file mmc3.pdf]

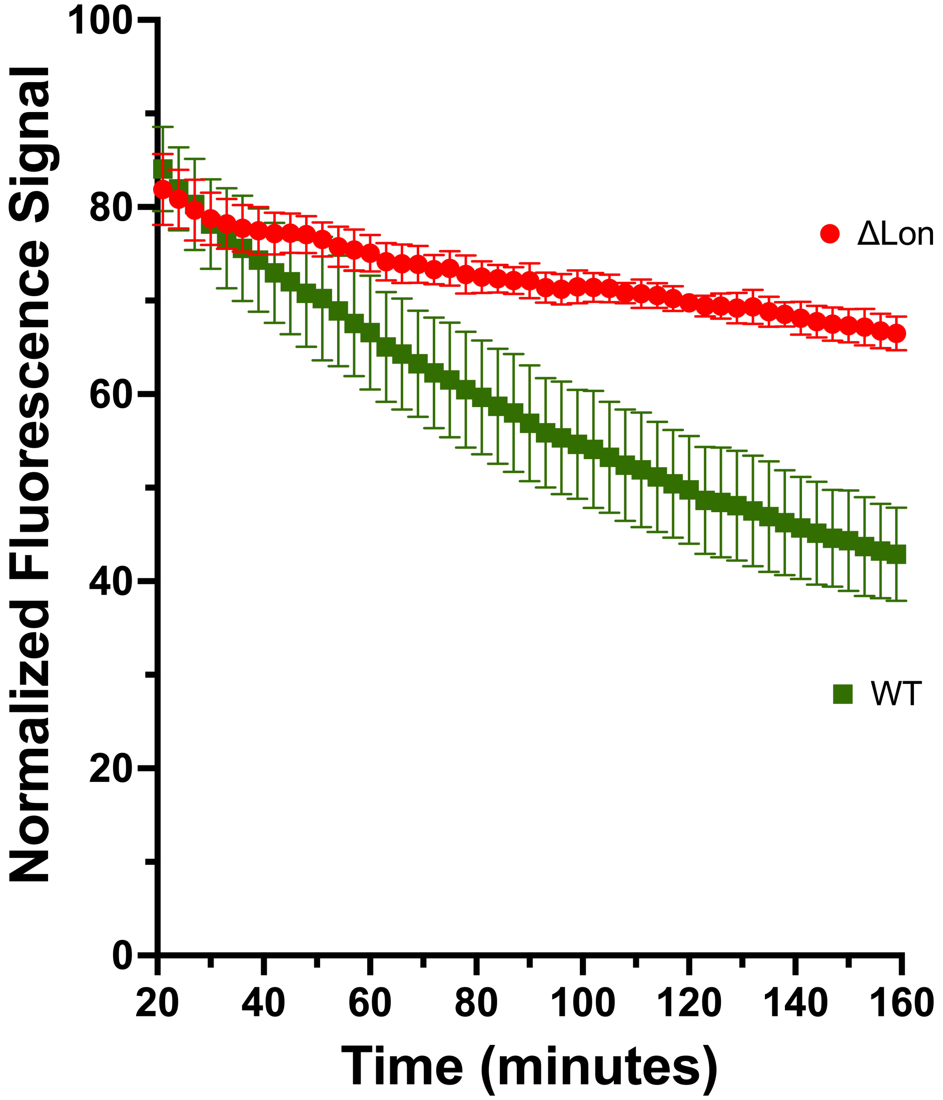

Fig. S1. GFP-0596 is markedly more stable in cells lacking Lon protease. In vivo degradation assay was carried out as described in the methods, and the fluorescence signal from the cell culture was recorded over time. The ΔLon cells do see a small decrease in signal, suggesting that there is minimal degradation of GFP-0596 by other proteases *in vivo*. However, the wildtype (Lon containing) cells exhibit a much larger loss in fluorescence over time, indicating that GFP-0596 is degraded much more readily in these cells.

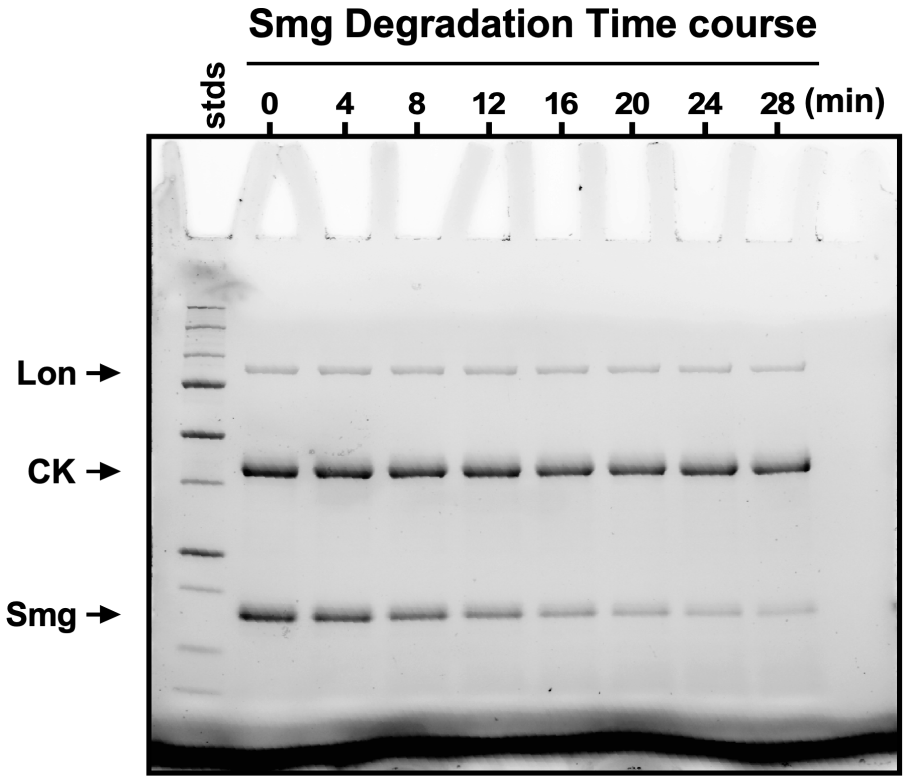

Fig. S2. ***In vitro*** **degradation of native Smg protein by Lon protease.** Native Smg protein carrying an N-terminal Hisx6 affinity tag was expressed and purified using a combination of Ni-NTA affinity, ion exchange, and size exclusion chromatography. Approximately 10 µM Smg protein was subjected to proteolysis by Lon protease in the presence of an ATP regeneration system. At the indicated time points, the reaction products were resolved by electrophoresis on a 15% tris-tricine gel and stained with Coomassie Brilliant BlueR250.

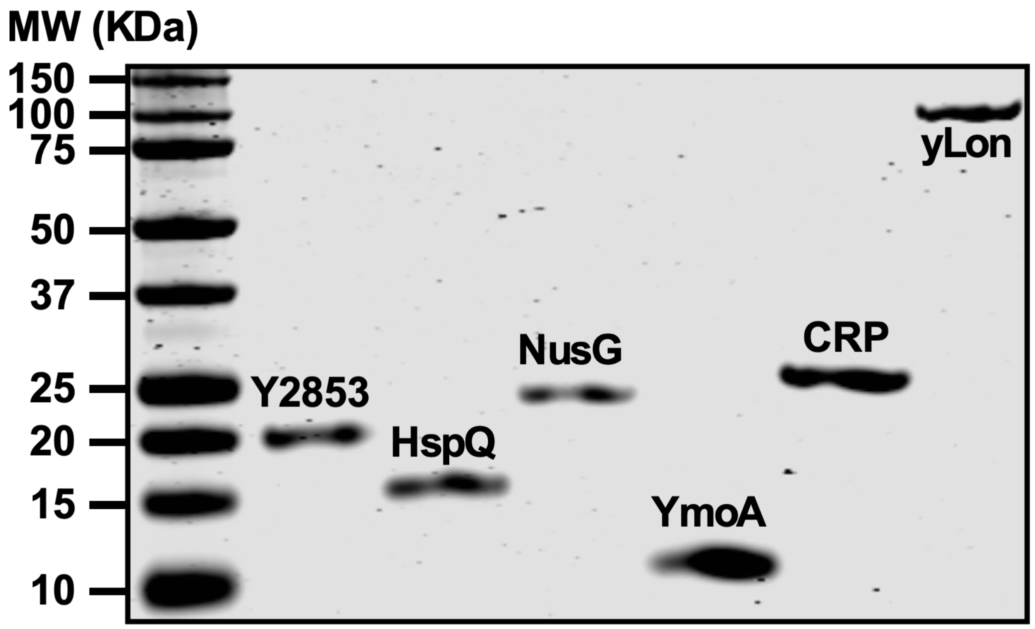

Fig. S3. Purified proteins for Lon proteolysis assays. Individual protein for Lon assays were cloned into overexpression plasmids with or without an N-terminal Hisx6 affinity tag. The proteins were individually expressed and purified using a combination of Ni-NTA affinity, ion exchange, and size exclusion chromatography. Approximately 1 µg of each protein was resolved by electrophoresis on a 15% tris-tricine gel and stained with Coomassie Brilliant BlueR250.
